# A 3-Axis Miniature Magnetic Sensor Based on a Planar Fluxgate Magnetometer with an Orthogonal Fluxguide

**DOI:** 10.3390/s150614727

**Published:** 2015-06-19

**Authors:** Chih-Cheng Lu, Jeff Huang

**Affiliations:** 1 Institute of Mechatronic Engineering, National Taipei University of Technology, Taipei 106, Taiwan; E-Mail: ffej918@hotmail.com; 2 Department of Mechanical Engineering, National Taipei University of Technology, Taipei 106, Taiwan

**Keywords:** 3-axis magnetometer, planar fluxgate, fluxguide, CMOS-MEMS

## Abstract

A new class of tri-axial miniature magnetometer consisting of a planar fluxgate structure with an orthogonal ferromagnetic fluxguide centrally situated over the magnetic cores is presented. The magnetic sensor possesses a cruciform ferromagnetic core placed diagonally upon the square excitation coil under which two pairs of pick-up coils for in-plane field detection are allocated. Effective principles and analysis of the magnetometer for 3-D field vectors are described and verified by numerically electromagnetic simulation for the excitation and magnetization of the ferromagnetic cores. The sensor is operated by applying the second-harmonic detection technique that can verify *V-B* relationship and device responsivity. Experimental characterization of the miniature fluxgate device demonstrates satisfactory spatial magnetic field detection results in terms of responsivity and noise spectrum. As a result, at an excitation frequency of 50 kHz, a maximum in-plane responsivity of 122.4 V/T appears and a maximum out-of-plane responsivity of 11.6 V/T is obtained as well. The minimum field noise spectra are found to be 0.11 nT/√Hz and 6.29 nT/√Hz, respectively, in X- and Z-axis at 1 Hz under the same excitation frequency. Compared with the previous tri-axis fluxgate devices, this planar magnetic sensor with an orthogonal fluxguide provides beneficial enhancement in both sensory functionality and manufacturing simplicity. More importantly, this novel device concept is considered highly suitable for the extension to a silicon sensor made by the current CMOS-MEMS technologies, thus emphasizing its emerging applications of field detection in portable industrial electronics.

## 1. Introduction 

Traditional fluxgate magnetic sensors typically possess high device sensitivity, low noise density and excellent sensing accuracy. However, they are still inferior to other magnetic sensors in the aspects of bulky volume of coils, higher power consumption and lower integration capability. Other competitive magnetometers such as anisotropic-magnetoresistance (AMR), tunneling magnetoresistance (TMR), giant-magnetoresistance (GMR), magnetoimpedence (MI) and magnetotransitor (MT) devices have been extensively studied and reported [1,2,3,4]. Nonetheless, with the progress of system miniaturization, the recent advance of miniature fluxgate sensors using CMOS-MEMS technologies has been promising. Fluxgate magnetometers are typically applied in craft navigation, military detection and medical recognition [5,6]. So far prospective applications of micro-fluxgates have been considerably developed for modern digital navigation [7], thoracoscopic surgery [8], and nondestructive inspection [9]. 

More importantly, recent development of fluxgate vector magnetometers has been brought to simultaneous 3-axis field detection methodology though such research cases are still relatively scarce when compared with general uni- and bi-axis fluxgate magnetometers. An interpretation method with the use of 3-axis fluxgate magnetometer array [10] had improved and compensated permanent and induced magnetic fields generated by magnetized objects and sensors. The mentioned method was a non-linear and inverse process that approximates three coordinates and the magnetization vector of a dipole. Later a series of efforts for the development of 3-axis ring cores and sensor design, tests and calibration procedure were implemented for spacecraft missions [11]; however, the 3-D ring core assembly rather than a planar structure may limit its wide applications in microelectronics or mobile systems. A typical procedure for building a 3-axis vector magnetometer is to align three uni-axis sensors along the three orthogonal sensing axes [12,13]. Although the assembly of such sensors is considered uncomplicated, it is still not feasible to achieve accurate alignment for individual sensors in a mass-production process. Recently, a number of works on 3-axis Lorentz force-based micro-electromechanical magnetometers have been reported [14,15], though this kind of devices usually have significant temperature dependence and limited measurement bandwidth.

On device characterization of fluxgate magnetometers, several earlier works [16,17] concluded that the sensing methodology and sensitivity enhancement by adopting the multiple harmonic frequency analysis techniques are practicable. In addition to magnetic core materials, design of excitation and pick-up coils is also critical to determine the functioning of a fluxgate device. Designs for planar excitation and pick-up coils [18,19] or three-dimensional (3-D) coils [20,21,22,23] were previously investigated. The traditional coils of fluxgate sensors, commonly wire-wound, are usually characterized as high sensitivity and low noise; but, they are still too bulky and system-incompatible to meet the dimensional requirements of a miniature product. To circumvent the drawbacks, micro-fluxgate sensors featuring planar CMOS pick-up coils and 3-D excitation coils with wire-bonded [24,25] and flip-chip [26] techniques were thus proposed and characterized. These classes of miniature fluxgate sensors were able to present useful negotiable solutions between device performance and inexpensive production processes. Moreover, a recent study on the field feedback techniques that improve linearity and stability of a tri-axial fluxgate sensor was also reported [27].

To realize a tri-axis magnetometer with undemanding fabrication process, an innovative approach using an orthogonal fluxguide to deflect the magnetic flux to the in-plane sensing directions of a vector GMR sensors has been recently reported by the authors’ group [28]. In this study, to develop a 3-axis micro-fluxgate device, we present a new design of a planar fluxgate magnetometer with a cross-shaped ferromagnetic core by using the fluxguiding concept. The miniature tri-axis fluxgate magnetometer is prototyped on a PCB substrate, analyzed by simulations and characterized by a series of experiments. Comprehensive characterizations such as responsivity investigation, field noise spectra, frequency response and low frequency geomagnetic field measurement, are also provided and discussed in this article. 

## 2. Design of the Fluxgate Magnetometer 

### 2.1. Tri-Axis Fluxgate Sensor with an Orthogonal Fluxguide

**Figure 1:**
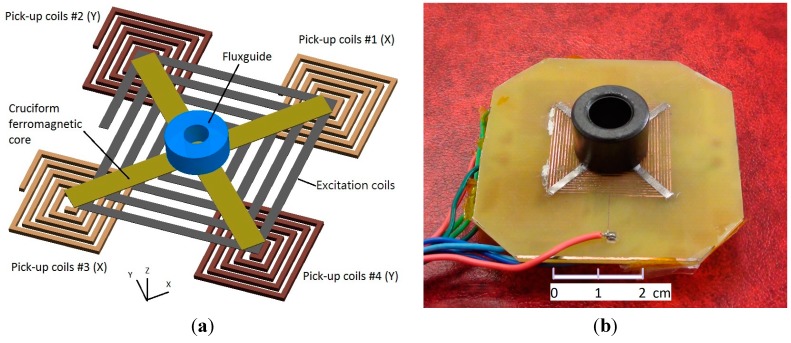
(**a**) Conceptual schematic of the planar fluxgate structure with an orthogonal fluxguide; (**b**) the PCB-based tri-axis fluxgate magnetometer. Note that all pick-up coils are implemented on the back side of the PCB.

The proposed tri-axis fluxgate sensor is mainly composed of a bi-axis fluxgate with a cruciform ferromagnetic core, a planar excitation coil and four planar pick-up coils. It is noted that the planar fluxgate in fact evolves from the Vacquier-type dual-core fluxgate magnetometer and combines two individual ferromagnetic cores in one, leading to a more compact configuration. The cross-shaped core is placed along the diagonals of the square excitation coils so that the excited magnetic fields are in opposite direction in pairs, and they can cancel each other in the pick-up coils, which are arranged beneath the excitation coils and centered on four endpoints of the cruciform core. Due to the nature of flux convergence (divergence) of the magnetic fields near the endpoints of the magnetic core, this design is able to detect in-plane magnetic field vectors in X- and Y-axis. To expand such a device to measure an additional magnetic field vector in the Z-axis, an orthogonally cylindrical ferromagnetic tube acting as a fluxguide is hence employed to collect or concentrate magnetic fields and then emit or deflect the fields into the planar cruciform core, as depicted in the perspective structure of Figure 1a. With the addition of a fluxguide, the bi-axial planar fluxgate sensor is able to detect the orthogonal field vectors along the Z-axis. The main advantage of the above design is beneficial from the much simpler process and assembly steps, which are rather dissimilar to those of certain spatial fluxgates fabricated or assembled by more complicated process [12,13]. The as-fabricated tri-axis fluxgate magnetometer is shown in Figure 1b.

Two diagonal pick-up coils are designed in series for each sensing direction such that the total number of turns for an individual axis is 24, and 20 for the excitation coils. The wire width of coils is 0.35 mm with a similar wire gap. The excitation coils are formed by photolithography and dip-etching processes on the front side of the PCB substrate, while four pick-up coils are made simultaneously on the back side. The square thick-film Cu wires of the planar coils configure with a thickness of 35 μm. Metglas™ 2714A ferromagnetic sheet (Metglas, Conway, SC, USA) is adopted as the ferromagnetic core and it is 15 μm thick, 2 mm wide and 40 mm long in each sensing direction, featuring a low magnetic saturation of 10 µT obtained by the second-harmonic analysis experiments. The cylindrical tube of Ni-Zn ferrite fluxguide is measured as 9 mm in inner diameter, 16 mm in outer diameter, and 13 mm in height. The permeability of the ferromagnetic core and ferrite fluxguide are 80,000 and 4π × 10^−4^ H/m, respectively. 

### 2.2. Design and Analytic Simulation of the Fluxgate Magnetometer

**Figure 2:**
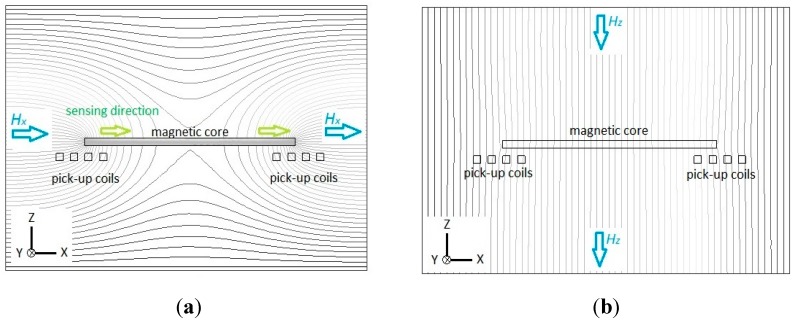
Flux line diagrams illustrate the sensing principle of the planar fluxgate only with a cruciform core and the cross-section is along the longitudinal core: (**a**) in X-axis sensing direction; (**b**) in Z-axis sensing direction.

To investigate the sensing principle of our proposed device, we utilize Maxwell^®^ SV 2-D simulator software (Ansoft Corporation., Pittsburgh, PA, USA, 2002) to model and analyze the visualization of magnetism. First, let us verify the sensing principle of the planar fluxgate only with a cruciform core, and the cross-section along the core is shown in Figure 2. For the detection of X-axis (or Y-axis) magnetic fields, the magnetic flux lines are assumed to be parallel to the axial direction of the magnetic core, as depicted in Figure 2a. It can be visibly seen that magnetic flux lines may exhibit convergence or divergence near the ends of the core, and more importantly, a number of flux lines are concentrated or squeezed into the interior of the core until they deviate into air (or vacuum) at the other end. This phenomenon helps generate a variation of the vector field intensity magnitude and flux density around both core ends and this thus can be converted to voltage induced by two pick-up coils beneath them. It is noted that the parallel flux lines near the up and bottom surface of the core may generate the same sensing directions for the corresponding pick-up coils in X-axis due to the fluxguiding (or flux concentration) effect. By implementing a X-Y sensing circuit, one can sum up the induced voltage of two pick-up coils and successfully obtain the vector magnitude of magnetic fields in X-axis (or Y-axis). On the other hand, the Z-axis flux lines, as observed in Figure 2b, are applied to pass through the magnetic core without any flux concentration effect occurred in it. Therefore the planar fluxgate is considered merely sensitive to the in-plane field vectors.

**Figure 3:**
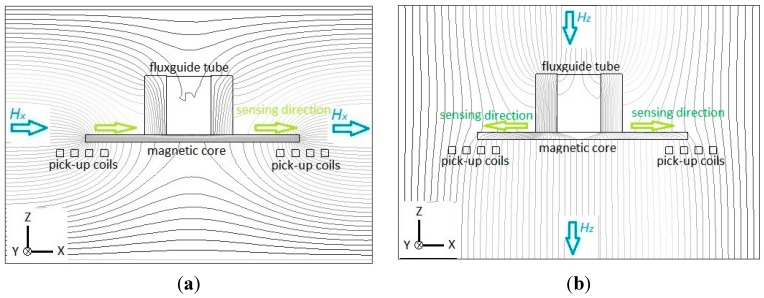
Flux line diagrams illustrate the sensing principle of the planar fluxgate with a cruciform core and an orthogonal fluxguide, and the cross-section is along the longitudinal core: (**a**) in X-axis sensing direction; (**b**) in Z-axis sensing direction.

Next, the working principle of the planar fluxgate with a cruciform core and a vertically orthogonal fluxguide is investigated. For the X-axis (or Y-axis) detection, the distribution of magnetic flux lines is considered highly comparable with the planar device, in particular near the ends of the core, when compared with Figure 2a and Figure 3a. The simply different part in the flux line diagrams is the interior region of the fluxguide in Figure 3a, which has a trivial effect on both ends of the core. Through the simulation of flux line distribution, it is confirmed that the tri-axis sensor with a fluxguide does not generate significant interference for X-axis (or Y-axis) detection and surely obtain the vector component of magnetic fields in X-axis (or Y-axis) as the planar one. In contrast to the same sensing directions for X-axis measurement, the sensing directions for pick-up coils are formed in reverse when exposed to Z-axis fields. Therefore, by employing a X-axis (or Y-axis) sensing mode with the same winding direction in the pick-up coils, one may sum up the induced voltage of two pick-up coils and successfully obtain the vector magnitude of magnetic fields in X-axis (or Y-axis) since the induction effect caused by the Z-axis fields is called off each other. On the other hand, for the measurement of the Z-axis field component, let’s assume that the magnetic fields are set to be parallel to the longitudinal orientation of the fluxguide. Figure 3b clearly reveals that considerable flux lines near the top of the fluxguide are bent and guided into its interior, and transferred in the magnetic core along X- axis (or Y-axis), and diverged into air (or vacuum) in the end. Similarly this phenomenon also generates a vector field intensity and flux density magnitude variation around both core ends and voltage is thus induced by two planar pick-up coils. As a result, it can be seen from Figure 3 that the sensing directions of two pick-up coils are transverse to the X-Y plane in the same direction when the sensor measures X-axis fields. On the other hand, the sensing directions of in-series pick-up coils are simutaneously outward (or inward) the center of the fluxguide while measuring the fields along Z-axis. By using Z-axis sensing mode with the opposite winding direction in pick-up coils, one may sum up the induced voltage from two or four planar pick-up coils and acquire the vector component of magnetic fields in the Z-axis direction. Likewise, the magnetic induction from the generated X- or Y-axis field vectors is finally called off in the pick-up procedure. In consequence, these can clarify that our proposed planar fluxgate with a fluxguide is sensitive to the orthogonal Z-axis field vectors. Hence, the field components in the X-, Y- and Z-axis are readily available if the orthogonal tri-axis sensing modes are properly applied and switched by the transducing circuit (to be discussed elsewhere). Nevertheless, comparing the magnetic flux density in the cores exhibited in Figure 3a,b, one can realize that the X-axis field components derived from the Z-axis field vectors are still far less than those from X-axis fields, and it may affect the responsivity of the Z-axis field direction. In addition, the experimental results of orthogonality analysis later exhibited in Table 1 are able to clarify the significant division of the spatial fields along the Cartesian coordinates. 

**Table 1:** The experimental results of orthogonality analysis.

	Condition	Under a X-Axis Field (50 G)	Under a Y-Axis Field (50 G)	Under a Z-Axis Field (50 G)	Non-Orthogonality (%)	
Measurement						
Pick-Up Voltage *V_x_* (X-axis sensing mode)		2.21 (V)	5.6 (mV)	16.8 (mV)	0.25 (X-Y plane)	0.76 (X-Z plane)
Pick-Up Voltage *V_z_* (Z-axis sensing mode)		16.4 (mV)	16.1 (mV)	204 (mV)	8.04 (X-Z plane)	7.89 (Y-Z plane)

A cross-cored fluxgate consists of two perpendicular ferromagnetic cores. If the permeability of the core material is assumed high, then the voltage in X-axis (or Y-axis) induced by these pick-up coils can be calculated on the basis of the Faraday’s law in consideration of demagnetization factor *D* as follows:

(1) $$V_{i}(t)=-2NA\frac{dB_{i}(t)}{dt}=-\frac{2NAH_{e}}{{\left(1+D\mu_{d}\right)}^{2}}\frac{d\mu_{d}}{dt}$$

where *N* is the number of each pick-up coil turns, *A* is the cross-sectional area of the magnetic core, *H_e_* is the external dc field, µ*_d_* is the time-dependent permeability and *B_i_*(*t*) is the magnetic flux density in the longitudinal course of the *i*-th magnetic core [26]. Equation (1) argues that the output voltage from all pick-up coils is associated with the number of coil turns *N*, external dc fields *H_e_* and the derivative of permeability µ*_d_*. Also, the time-dependent permeability µ*_d_* is comparable to the gradient of the *B-H* curve of the core material, and in consequence the higher it is, the greater the output voltage is. Besides, demagnetization factor *D* is considered dominant in building up the induced voltage. As one knows, demagnetization factor is mainly related to the aspect ratio of core diameter (or width in this study) to core length. Accordingly, a narrow and long magnetic core featuring a smaller aspect ratio can considerably reduce demagnetization effect and thus raise the voltage in Equation (1). Apart from the use of a high-permeability core, it is also essential to optimize the dimensional configurations of the planar coils and the magnetic core for responsivity enhancement and power reduction in consideration of demagnetization effects.

### 2.3. Simulation and Analysis for Excitation Coils and Core Magnetization 

**Figure 4:**
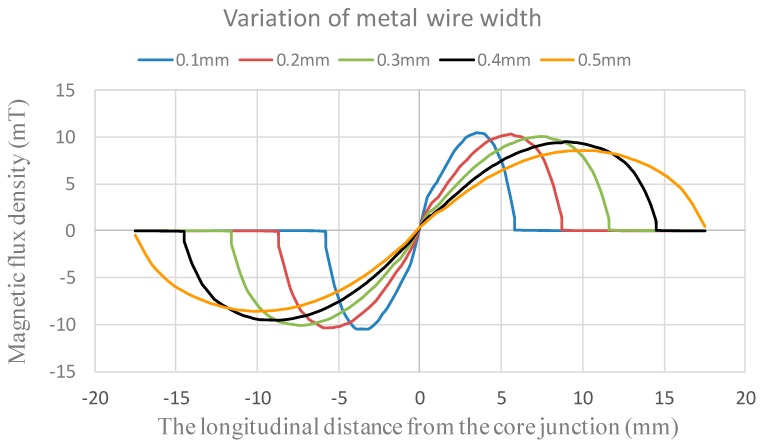
The theoretical magnetic flux density along the magnetic core *vs.* the distance from the core junction with respect to various wire width.

Furthermore, by using an electromagnetic 3-D simulator ANSYS Maxwell^®^ (ANSYS, Inc., Canonsburg, PA, USA), it is imperative to find the locally optimal dimension in terms of the metal wire width of the excitation coils for the proposed fluxgate. Therefore, we simulate and evaluate the distribution of magnetic flux density (*B*) along the axis of the magnetic core while core magnetization and sensor excitation are fulfilled. To investigate design diversity of the copper excitation coils, the following parameters are modulated for simulations: (1) metal wire width = 0.1–0.5 mm with a 0.1 mm interval; (2) metal wire gap = 0.1–0.5 mm with a 0.1 mm interval; and (3) excitation current = 0.1–0.5 A with a 0.1 A interval. Next, some key constants and variables are given: (1) Cu metal resistivity = 17 μΩ-m; (2) core (2714 A) permeability = 80,000 H/m; and (3) core length (single rod) = 40 mm and core width = 2 mm. It can be seen that numerous simulations were carried out by a combination order, and one can acquire the induction trend excited by the excitation coils. For example, assuming a uniform excitation current through the coils, one of the series simulation results provided in Figure 4 indicates the theoretical magnetic flux density along the core length with respect to various Cu metal wire width from 0.1 mm to 0.5 mm when metal wire gap is 0.1 mm and excitation current is 0.5 A. From the comprehensive simulations, it is reveled that the maximum value of magnetic flux density appears to occur near the middle position of the half-core, and the influence of various metal wire gaps on the magnetic flux density can be trivial. Thus, by considering the overall sensor dimensions and PCB processing feasibility, the miniature fluxgate is fabricated to have an excitation coils with 0.35 mm in wire gap as well as wire width for further device analysis later. 

**Figure 5:**
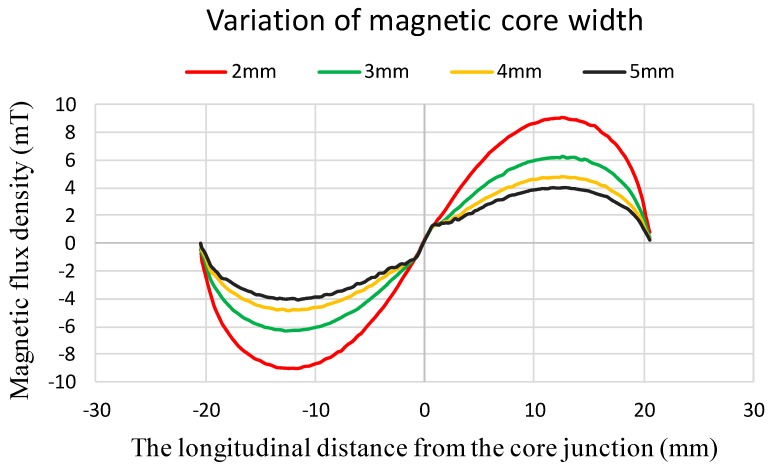
The theoretical magnetic flux density along the longitudinal core *vs.* the distance from the core junction with respect to various core width.

Also, based on the resultant parametric settings, we survey magnetic flux density along the longitudinal distance of the magnetic core *vs.* various ferromagnetic core width designs. Therefore, as shown in Figure 5, its shown that the theoretical magnetic flux density in the interior of core along the longitudinal direction is relatively decreased when the core width is increased from 2 mm to 5 mm. Apparently, the variation of core width is related to the cross-sectional area and demagnetization effect of the core geometry, and by referring to Equation (1), the simulation results also make it evident that the demagnetization factor is probably more dominant in improving the device responsivity.

**Figure 6:**
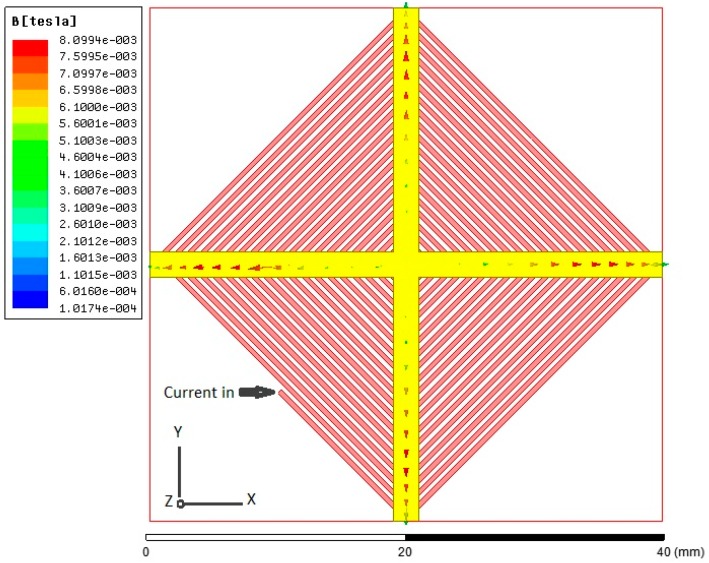
A 3-D model of a planar excitation coils and its simulated result with a 2-mm cruciform ferromagnetic core under the excitation current of 5 A.

**Figure 7:**
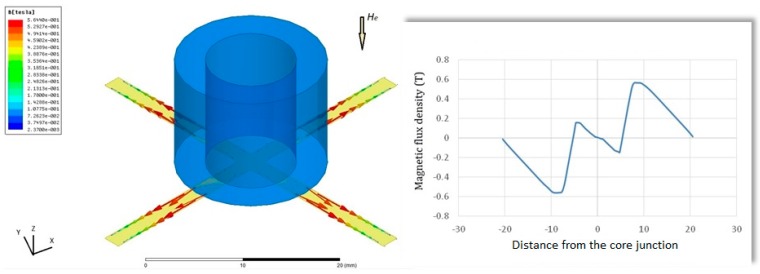
A 3-D modeling and simulation result of a tri-axis planar device with a 2-mm cruciform ferromagnetic core and a fluxguide (**left**); The variation of magnetic flux density along the core is also available and the magnetic fields in Z-axis is 40 A/m (**right**).

Afterward, a 3-D model example of the planar excitation coils with a 2-mm cruciform ferromagnetic core, as shown in Figure 6, is accordingly built and analyzed. For electromagnetic excitation and core magnetization, an excitation current of 0.5 A and the *B-H* loop data of the core material are employed, and it is found that the excitation coils are easily able to achieve the magnetic saturation of the core employed (>10 µT). Again the location of maximum flux density is approximately close to the middle position of the half-core in X- or Y-sensing orientation, which is feasible for in-plane detection. In addition, to measure the orthogonal Z-axis fields, another ultimate design including the planar excitation coils with a cruciform core and a ferrite fluxguide tube, as given in Figure 7, is then analyzed under a 40 A/m magnetic field along Z-axis. One can observe that a linear distribution of magnetic flux density in the core rises from the inner diameter (*i.e.*, ±4.5 mm) to the outer diameter (*i.e.*, ±8 mm) and reaches the maximum value of 0.56 T, yet, beyond that point, the magnetic flux density is linearly decreased to zero with the increased longitudinal distance till the end of the core. The orthogonal flux vectors guided into the in-plane core, even though not all of them, can be easily measured by switching to the Z-axis sensing mode. According to the simulated result, it is also promising to elevate the variation of magnetic flux density, *i.e.*, the responsivity, within the sensing area of a pick-up coils simply by placing a larger cylindrical fluxguide tube close to the end of the core. From the manufacturing point of view, the proposed simple structure is particularly considered superior to those complicated 3-D devices using traditionally monolithic or assembly methods employed before.

## 3. Device Characteristic Results 

**Figure 8:**
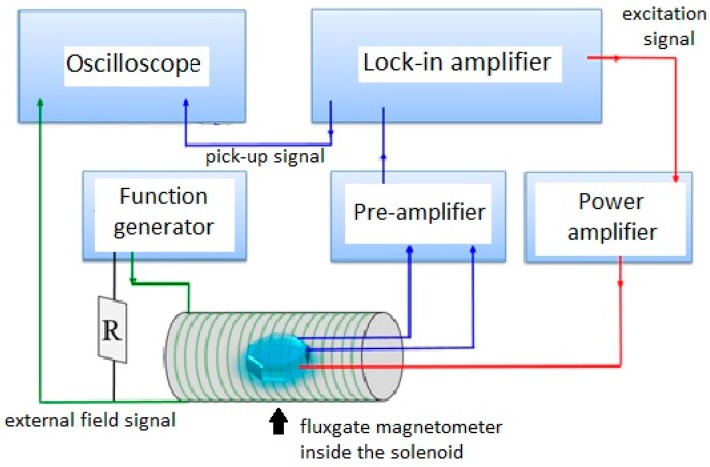
A schematic diagram of the fluxgate magnetometer setup for characterization measurement.

Device characteristics of the proposed fluxgate sensor are implemented by a measurement system shown in Figure 8. The system is able to perform the second-harmonic detection and generate external magnetic fields from an ac modulated solenoid. Basically the measurement system includes a power amplifier (BA4825, NF Corp., Yokohama, Japan), a lock-in amplifier (SR830, Stanford Research Systems, Sunnyvale, CA, USA), a signal pre-amplifier (SR560, Stanford Research Systems), a function generator and a digital oscilloscope. In practice, the second harmonics are generated from the planar pick-up coils and exhibited by the lock-in amplifier using a phase-reference method. Therefore, the sensitivity, or known as responsivity *(dV*/*dB)*, of the fabricated magnetometer can be fully defined and evaluated by recording the magnitudes of the pick-up voltage and magnetic fields via these instruments.

### 3.1. Responsivity Measurement of the Fluxgate

**Figure 9:**
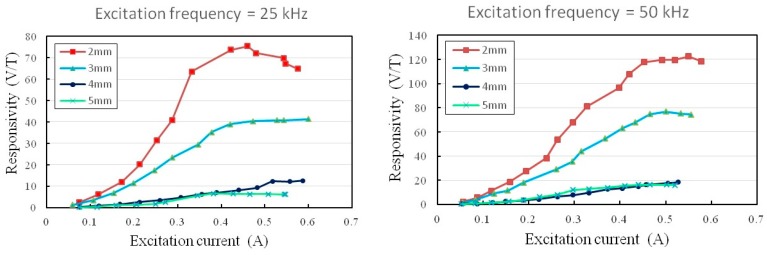
In-plane sensor responsivity (e.g., X-axis) *vs.* the excitation current at 25 kHz and 50 kHz, respectively.

As reported by several previous studies [16,17,25], the main electrical parameters such as the amplitude and frequency of the excitation current can modulate the responsivity of the fluxgate device in effect. The amplitude modulation on the responsivity of X-axis (or Y-axis) is first carried out. The relationship between the responsivity (*dV*/*dB*) and the excitation current at 25 kHz and 50 kHz of excitation frequencies with respect to different core width is explored, respectively, and shown in Figure 9. The responsivity is acquired by analyzing the sensor’s second harmonic signals respecting to different sets of the core width (*i.e.*, 2 mm, 3 mm, 4 mm and 5 mm) under a constant excitation frequency. Based on the previous discussion in Section 2.2 and Section 2.3, it is evident that the ferromagnetic core with a smaller aspect ratio (*d*/*L*) can decrease demagnetization effect and contribute a significant rise of the pick-up voltage for responsivity. For instance, the field responsivity in X-axis, by using 2 mm of core width and 25 kHz of excitation frequency, could arrive at the maximum value of 75.4 V/T at 460 mA of current amplitude, and finally made a descent to 64.8 V/T at 580 mA of the current limit. Similarly, with 2 mm of core width under the high excitation frequency of 50 kHz, the maximum responsivity of 122.4 V/T was stably obtained and saturated prior to 575 mA. These measurement results hence verify the responsivity characteristics of in-plane axes (*i.e.*, X- or Y-axis).

One of our previous studies [25] revealed that the excitation current may be reduced in respect of rising excitation frequency. The reduction of excitation current can probably be due to the impedance increase of the excitation coil. Yet the impedance change of the L-R circuit is found minor (~0.1%) up to 100 kHz, and thus it’s suggested that the increase of impedance may result from the skin effect in measurement rather than the inductance change of the coils. It has also been demonstrated [26] that the field-to-voltage transfer coefficient generally ascends with the increasing amplitude of excitation current and rising excitation frequency. Besides, one can observe an obvious *dV*/*dB* decline on the curve of 2-mm core width under 25 kHz when the excitation current exceeds 460 mA, and this can be possibly attributed to the deterioration effect of the second harmonic and the enhancement of other even harmonics together. Another similar experimental result obtained from a chip-scale fluxgate was revealed and discussed in [25]. Therefore, the sensor’s responsivity can be further advanced while the excitation frequency goes up. However, a high-frequency operation may considerably increase circuitry complexity and instrumentation cost of the sensor system.

**Figure 10:**
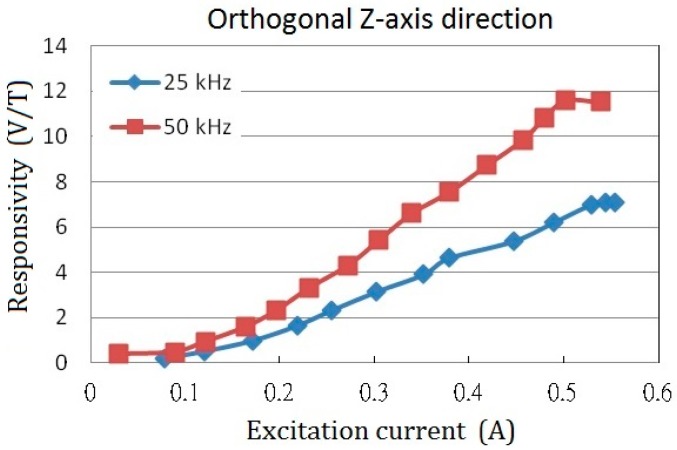
The orthogonal sensor responsivity (*i.e.*, Z-axis) *vs.* the excitation current at 25 kHz and 50 kHz excitation frequencies, respectively.

Likewise, one can be interested in the responsivity of the orthogonal Z-axis with a 2-mm wide core. The responsivity in Z-axis against the excitation current with regard to different excitation frequencies are shown in Figure 10. It can be seen that the maximum values of the Z-axis responsivity are 7.1 V/T and 11.6 V/T at 25 kHz as well as 50 kHz, respectively, and obviously they are much smaller than those obtained in X-axis (or Y-axis). This is not unusual because the X-axis (or Y-axis) magnetic fields (*H*) derived from the Z-axis orientation is far less than the fields distribute in Z-axis. Nevertheless, it is proven to successfully detect the magnitude of the orthogonal magnetic field vector through the planar structure with a perpendicular fluxguide. We also compare the fluxguiding effect between the solid and tubular cylinders as the fluxguides and the results are rather comparable. However, the use of a tubular fluxguide is advantageous for cost- and weight-reduction of the fluxguide component. 

### 3.2. Orthogonality Analysis of the Axes

To further investigate the magnetostatic coupling effect of the tri-axis device, experiments using a 0.5-A excitation current at 50 kHz excitation frequency are applied in sequence to the independent X-, Y-, and Z-axis orientation so as to verify the axial orthogonality of the fluxgate magnetometer. The orthogonality analysis was carried out in a calibration system made of a tri-axis Helmhortz coil and a precision stage where the sensor is mounted. Via the calibration system, e.g., the alignment accuracy of the field axes with respect to the corresponding in-plane pick-up coils and the fluxguide is smaller than 1°, and the alignment accuracy of the tri-axis Helmhortz coil is also assured within 1°. The experimental results were listed and analyzed in Table 1. As can be seen, the axis-coupling effect, or termed non-orthogonality, while running the X-axis sensing mode with respect to Y- and Z-axis is as low as 0.25% and 0.76%, indicating the exceptional orthogonality among the axes of the magnetometer. Similarly, the axis-coupling effect when running the Z-axis sensing mode with respect to X- and Y-axis becomes significant as 8.04% and 7.89%, respectively. These undesirable effects may result from poor orthogonality between axes due to fabrication or assembly steps. Also, using the material with higher permeability for both ferromagnetic cores and fluxguide is considered effective to diminish non-orthogonality of the proposed magnetic sensor. Alternatively, it is necessary to further explore the geometric design of the ferrite fluxguide or re-consider the layout of the pick-up coils as to improve the orthogonal responsivity in Z-axis. 

### 3.3. Noise Measurement and Frequency Response

**Figure 11:**
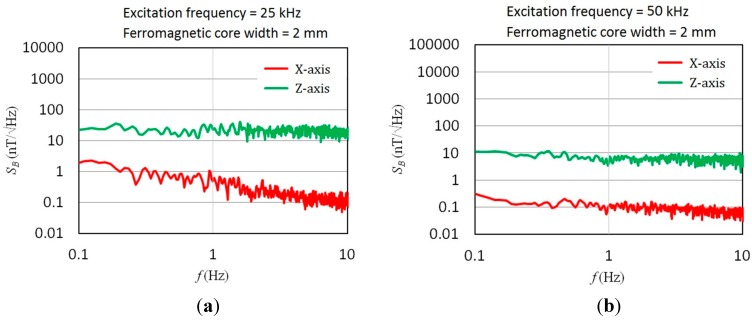
Field noise spectra of the magnetometer with 2-mm core width under different excitation frequencies in X- and Z-axis: (**a**) at 25 kHz; (**b**) at 50 kHz.

In addition to responsivity characterization, field noise analysis of the micro-fluxgate sensor under a magnetically shielded environment is also vital to assess the feasibility of a magnetometer. The field noise spectra was measured under a magnetically shielded environment by using a lock-in amplifier and a power amplifier to generate excitation current for the fluxgate sensor, and adopting a spectrometer to measure the voltage noise *vs.* frequency from 0.1 Hz to 10 Hz. The field noise is defined as the ratio of the voltage noise to the responsivity under specific excitation voltage. All noise measurements were implemented with core width of 2 mm under 25 kHz and 50 kHz, respectively. As shown in Figure 11, it is evident that the field noise density descends while the excitation frequency increases from 25 kHz to 50 kHz, which may help trim down field noise spectra at low-frequency fields (<10 Hz). As a result, experiments shown in Figure 11a reveal that the minimum field noise spectra in the X-axis direction observed at 1 Hz of magnetic fields were 0.53 nT/√Hz under a 25 kHz excitation and 0.11 nT/√Hz under a 50 kHz excitation, correspondingly. Likewise, as shown in Figure 11b, the lowest field noise spectra in the Z-axis direction at 1 Hz of magnetic fields were measured as 21.91 nT/√Hz and 6.29 nT/√Hz under 25 kHz and 50 kHz of excitation frequency. In fact, in the relatively low frequency bandwidth (~10 Hz), one can observe that the magnetic sensor demonstrates a relatively improved field noise density against the frequency variation. In addition, by taking Figure 9, Figure 10 and Figure 11 into account, it is noteworthy that the responsivity values of X- and Z-axis measured at 25 kHz and 50 kHz differ by a factor of 10-fold, and the field noise values of X- and Z-axis, for example at 1 Hz, differ by about a factor of 40–60 folds with respect to 25 kHz and 50 kHz, respectively. This fact implies that an additional factor of 4–6 fold is introduced then. We thus consider the reinforced effect in the field noise could be attributed to the following reasons: (1) different gain constants may be employed for the X-axis and Z-axis voltage noise measurements; and (2) the correlation between the field noise induced by the magnetic core and the orientation of excitation fields. For instance, the sensing direction of in-series pick-up coils while running the X-axis sensing mode may reduce portion of the voltage noise. In contrast, the sensing direction of in-series pick-up coils while running the Z-axis sensing mode may amplify the voltage noise in parts. These facts may have a partial influence on the field noise we measured.

**Figure 12:**
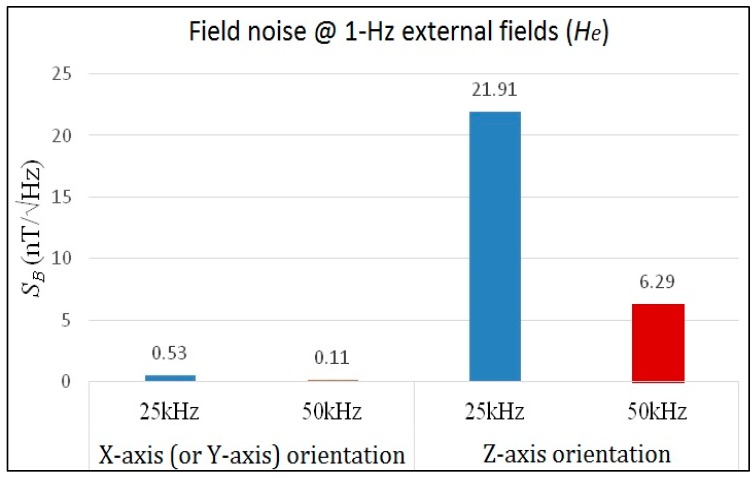
Comparison of the maximum field noise spectral density results under different excitation frequencies in X- and Z-axis.

Therefore, as illustrated in Figure 12, it is noteworthy that the field noise characteristics between the orthogonal axes are remarkably distinct. Apparently, the low field noise of the proposed sensor in X-axis (or Y-axis) can reach a resolution of sub-nT/√Hz. One can attribute this to the planar structure that features satisfactory responsivity and low voltage noise. However, the field noise density measured in the Z-axis direction is found to be approximately 1–2 orders greater when compared with that in the X-axis direction at the same excitation frequency, and this is possibly caused by an increased voltage noise density and poorer Z-axis responsivity. In addition, the design of low-noise CMOS circuits can be also helpful to decrease the voltage noise density and improve device detectivity. Moreover, some early developed PCB-based fluxgate devices [18,19,29] were typically regarded as more enhanced than the MEMS micro-fluxgates in both responsivity and field noise density because of their considerable dimensions.

**Figure 13:**
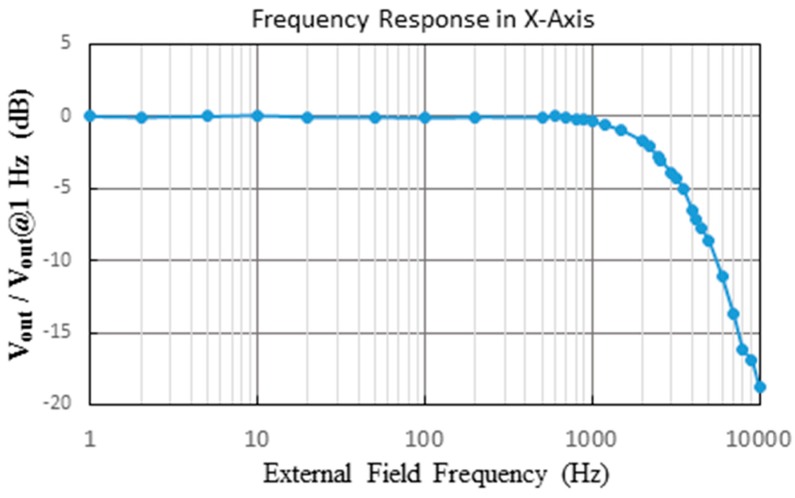
The frequency response result of the fluxgate with regard to the external field frequency.

Moreover, to explore the feasible operation range of the fluxgate sensor in terms of ac frequency of external magnetic fields, the frequency response of the device is thus analyzed. One can clearly see, as depicted in Figure 13, that the proposed tri-axis fluxgate magnetometer is able to detect a static dc or a low-frequency ac magnetic fields under 800 Hz, and beyond that point, the sensor’s responsivity is expected to decline rapidly. 

### 3.4. Linearity Characterization and Geomagnetic Fields Detection

In our previous work we investigated the second harmonic *dV*/*dB* at various frequencies and concluded that the sensor’s responsivity is highly affected by excitation frequency. The reduction in *dV*/*dB* at a higher excitation frequency, e.g., greater than 110 kHz, can be explained by the reduced excitation current amplitude at the same time [16,17]. When the materials of the magnetic cores are identical, it is found that the frequency of the pick-up voltage is equivalent to the second harmonics of the excitation signal. Thus, if the external fields are insignificant, according to the definition of Equation (1), the peak amplitude of the pick-up voltage is almost proportional to the external fields *H_e_*. 

**Figure 14:**
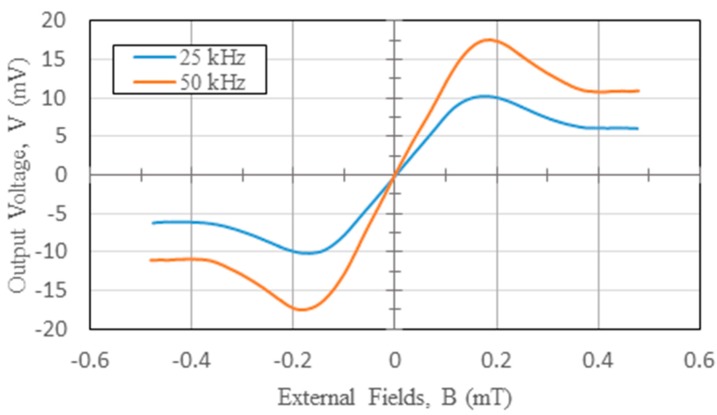
The V-B diagram close to zero magnetism at different excitation frequencies in X-axis.

To further explore the linear detection range, we observe the *V-B* correlation in the vicinity of the zero-magnetism to examine the sensor’s linearity. For instance, the *V-B* measurements comprising in-phase and quadrature-phase results using the second harmonic operation can well explain the liner range of the magnetic sensor. From Figure 9, it was shown that the corresponding responsivity of 74.1 V/T in X-axis, which was measured at an excitation current of 550 mA, with a core width of 2 mm, and under an lower excitation frequency of 25 kHz, could result in an excellent linear detection range of ±0.15 mT based on the linearity error of 10%, as shown in Figure 14. Since terrestrial magnetism ranges from 0.03 mT to 0.06 mT, the fluxgate magnetometer is thus considered highly feasible for electronic compasses or other advanced applications.

**Figure 15:**
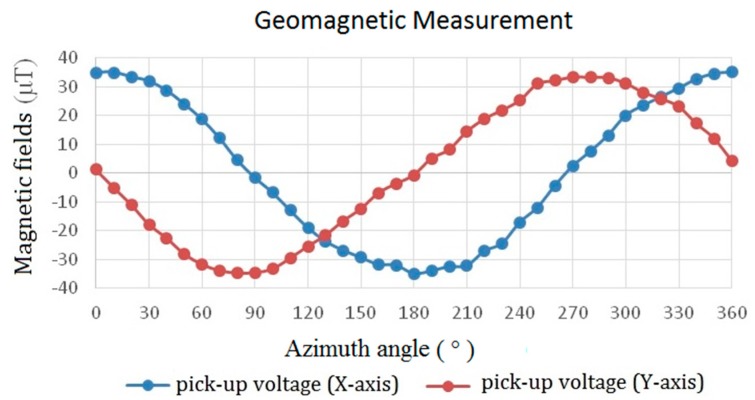
The geomagnetic measurement results of the planar fluxgate magnetometer as an electric compass under a excitation frequency of 25 kHz.

**Figure 16:**
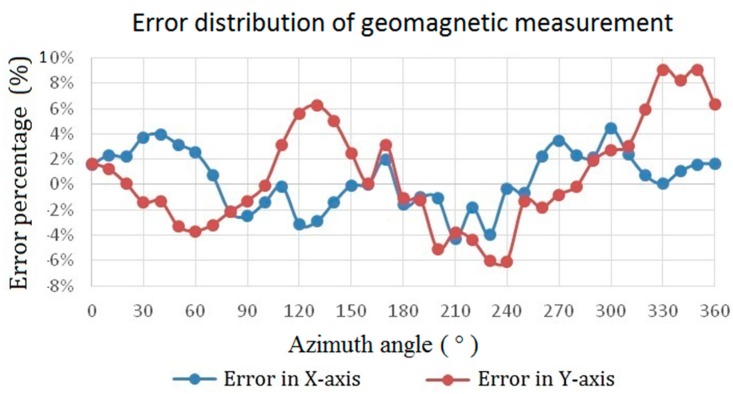
The error distribution of geomagnetic measurement for the in-plane axes with respect to the azimuth angle.

To verify dc or low frequency (~1 Hz) characteristics of the proposed device, it is further employed as an electronic compass to detect geomagnetic fields in the laboratory by carrying out an in-plane measurement, as demonstrated in Figure 15. As can be seen, the measured magnitude of geomagnetic fields corresponding to the azimuth angle in both the X and Y axes are highly consistent and reveal an accurate angle difference of 90° between two in-plane axes. If we calculate the error percentage of the magnetometer by using the theoretically spatial vectors of earth fields, the distribution of measurement error in planar axes with respect to azimuth angle can be obtained in Figure 16 where it exhibits the error distribution within a small range of ±10%. As a result, these favorable results provided in the work verify the promising characteristics of the planar fluxgate magnetometer by using the innovative design of an orthogonal fluxguide.

## 4. Concluding Remarks 

An innovative tri-axis design to measure the spatial vectors of dc or low-frequency ac magnetic fields by employing a miniature planar fluxgate magnetometer with an orthogonal fluxguide is presented. The proposed device structure utilizes an uncomplicated method to realize a miniature fluxgate developed on the PCB substrate. Simulations and numerical analyses for the key dimensions of the sensor to obtain good responsivity are carried out. In addition, comprehensive investigations on the device characteristics including responsivity modulation, field noise spectral density, linear range characterization and geomagnetic fields detection are also provided and discussed. The proposed magnetic sensor has maximum responsivity values of 122.4 V/T along X-axis (or Y-axis) and 11.6 V/T along orthogonal Z-axis respectively under 50 kHz of excitation frequency. The minimum magnetic field noise responses are 0.11 nT/√Hz in the X-axis direction and 6.29 nT/√Hz in the Z-axis direction when both are under 1 Hz of external fields. In addition, the in-plane measurement of the geomagnetic fields is successfully demonstrated. In the future outlook for the fluxgate magnetometer, experimental outcomes in this study may provide a promising foundation to develop chip-scale CMOS-MEMS micro-fluxgate devices for their more emerging applications. 
